# Free Water in White Matter Differentiates MCI and AD From Control Subjects

**DOI:** 10.3389/fnagi.2019.00270

**Published:** 2019-10-02

**Authors:** Matthieu Dumont, Maggie Roy, Pierre-Marc Jodoin, Felix C. Morency, Jean-Christophe Houde, Zhiyong Xie, Cici Bauer, Tarek A. Samad, Koene R. A. Van Dijk, James A. Goodman, Maxime Descoteaux, Alzheimer's Disease Neuroimaging Initiative

**Affiliations:** ^1^Imeka Solutions, Inc., Sherbrooke, QC, Canada; ^2^Sherbrooke Connectivity Imaging Lab, University of Sherbrooke, Sherbrooke, QC, Canada; ^3^VITAlab, University of Sherbrooke, Sherbrooke, QC, Canada; ^4^Digital Medicine & Translational Imaging, Early Clinical Development, Pfizer Inc., Cambridge, MA, United States; ^5^Department of Biostatistics and Data Science, The University of Texas Health Science Center at Houston, Houston, TX, United States; ^6^Internal Medicine Research Unit, Pfizer Inc., Cambridge, MA, United States

**Keywords:** Alzheimer disease, diffusion MRI, free water, neuroinflammation, white matter hyper intensity, mild cognitive impairment, white matter, diffusion tensor imaging

## Abstract

Recent evidence shows that neuroinflammation plays a role in many neurological diseases including mild cognitive impairment (MCI) and Alzheimer's disease (AD), and that free water (FW) modeling from clinically acquired diffusion MRI (DTI-like acquisitions) can be sensitive to this phenomenon. This FW index measures the fraction of the diffusion signal explained by isotropically unconstrained water, as estimated from a bi-tensor model. In this study, we developed a simple but powerful whole-brain FW measure designed for easy translation to clinical settings and potential use as a priori outcome measure in clinical trials. These simple FW measures use a “safe” white matter (WM) mask without gray matter (GM)/CSF partial volume contamination (*WM*_safe_) near ventricles and sulci. We investigated if FW inside the *WM*_safe_ mask, including and excluding areas of white matter damage such as white matter hyperintensities (WMHs) as shown on T2 FLAIR, computed across the whole white matter could be indicative of diagnostic grouping along the AD continuum. After careful quality control, 81 cognitively normal controls (NC), 103 subjects with MCI and 42 with AD were selected from the ADNIGO and ADNI2 databases. We show that MCI and AD have significantly higher FW measures even after removing all partial volume contamination. We also show, for the first time, that when WMHs are removed from the masks, the significant results are maintained, which demonstrates that the FW measures are not just a byproduct of WMHs. Our new and simple FW measures can be used to increase our understanding of the role of inflammation-associated edema in AD and may aid in the differentiation of healthy subjects from MCI and AD patients.

## 1. Introduction

White matter (WM) atrophy in Alzheimer's disease (AD) was observed more than three decades ago (Brun and Englund, 1986a). The microstructural changes observed in the WM of AD patients include axonal deterioration, Wallerian degeneration, loss of myelin density, loss of oligodendrocytes, microglia activation, and vascular degeneration (Brun and Englund, 1986b; de la Monte, 1989; Brilliant et al., 1995; Englund, 1998; Burns et al., 2005; Sjöbeck et al., 2005). Numerous studies have shown that changes in the WM are an early event in the development of AD, happening in preclinical stages (de la Monte, 1989; Kantarci et al., 2005; Desai et al., 2009). Changes in the microstructure of WM have even been reported before measurable hippocampal atrophy in mild cognitive impairment (MCI) (Zhuang et al., 2013) and preclinical AD (Hoy et al., 2017). More recent evidence shows that chronic neuroinflammation also contributes to the process of neurodegeneration in AD and was recently observed in the WM of AD patients (Raj et al., 2017).

Microglia-induced neuroinflammation in patients has been mostly studied using PET imaging ligands such as [11C]-PK11195 (Zimmer et al., 2014). However, to identify WM changes, diffusion MRI has been the modality of choice (Jones, 2010). Studies in the past decade have identified various regions in the WM where diffusion measures, mostly diffusion tensor imaging (DTI)-based measures such as fractional anisotropy and mean, axial, and radial diffusivities, correlate with symptoms of MCI and AD (Stebbins and Murphy, 2009; Smith et al., 2010; Nowrangi and Rosenberg, 2015; Galluzzi et al., 2016; Mito et al., 2018). A more recent diffusion measure is the free water (FW) index, which measures the fraction of the diffusion signal explained by isotropically unrestricted water (Pasternak et al., 2009), as estimated from a regularized bi-tensor model. In white matter, this measurement represents either FW in extracellular space around axons or FW contamination from cerebrospinal fluid in adjacent voxels. An elevated FW index in white matter has been suggested to indicate neuroinflammation (Pasternak et al., 2012a) and has been described in normal aging (Chad et al., 2018) and many neurological disorders such as schizophrenia (Pasternak et al., 2012b, 2015, 2016), Parkinson's disease (Ofori et al., 2015), and AD (Maier-Hein et al., 2015; Ji et al., 2017; Montal et al., 2018).

Association between higher FW, worse scores on a clinical dementia rating (CDR) and higher probability to transition to a more severe CDR stage was recently demonstrated by Maillard et al. (2018). In AD and MCI patients, an association between the widespread increased FW and poorer attention, executive functioning, cognitive performance, visual construction, and motor performance supports the idea that FW metrics are associated with clinical symptoms (Ji et al., 2017; Montal et al., 2018; Reas et al., 2018). In addition, DTI measures that have undergone correction for FW content have been shown to be more sensitive in differentiating between AD patients with and without cerebrovascular involvement compared to standard DTI measures (Ji et al., 2017). In a longitudinal study, FW-corrected radial diffusivity, but not un-corrected radial diffusivity, was higher in the WM of MCI patients who converted to AD compared to MCI patients who did not convert (Maier-Hein et al., 2015). FW-corrected DTI measures also demonstrate greater sensitivity to associations between AD pathology and white matter microstructure compared to standard DTI measures (Hoy et al., 2017).

Based on the growing body of evidence showing the association of FW or FW-corrected metrics with clinical symptoms of AD and concomitant cerebrovascular disease, we set out to develop a single powerful FW measurement that is easily translatable to clinical settings with potential to be used as a priori outcome measure in clinical trials. Simple volume-based measurements such as ventricular expansion, cortical/sub-cortical gray matter atrophy and WMH volume have been shown to be linked with various AD symptom but none of them gives information on normal appearing white matter, which may be affected earlier during transitional stages of normal aging to MCI and AD.

When measuring FW in aged subjects one needs to take into account white matter lesions that are visible on certain structural MR scans as white matter hyperintensities (WMHs). The number and total volume of WMHs are known to increase with age (de Leeuw et al., 2001) and they have been associated with vascular disease (Debette et al., 2010), cognitive impairment (DeCarli et al., 2001; Yoshita et al., 2006), and even directly with AD (Kandel et al., 2016). Since FW inside WMHs is very high compared to the subtle FW changes specific to the AD continuum, WMHs need to be removed to adequately measure AD specific FW changes. WMHs and FW are known to be part of a WM injury continuum (Maillard et al., 2017) and WM in the WMHs surrounding area (called the penumbrae) is also know to undergo microstructural changes (Maillard et al., 2011, 2014). To keep any WMHs related signal out of our FW measurement, a dilated version of the WMHs covering the estimated range of the penumbrae (Maillard et al., 2011) is removed from the final mask. Another pitfall is that due to the generally lower spatial resolution of diffusion images, partial volume contamination from sulci and ventricular CSF can considerably boost FW values leading to incorrect FW measurements. Some studies (Ji et al., 2017) avoid partial volume effects by using Tract-Based Spatial Statistics(TBSS; Smith et al., 2006). This method avoids the partial volume effect by projecting the data on a WM skeleton but has some shortcomings (Bach et al., 2014) that we want to avoid such as the loss of a major part of the WM voxels and atlas registration. In order to get an unbiased and relevant measure of FW in healthy WM, we developed a WM “*safe”* mask (*WM*_safe_) minimizing GM/CSF partial volume contamination and thus avoiding the shortcomings of TBSS.

In this study, we developed these simple yet powerful whole-brain FW measures without tractography or atlas registration. These measurements can be done on low angular resolution diffusion images and are designed for clinical settings and potential use as a priori outcome measure in clinical trials. This was done by designing a FW processing pipeline that computes whole-brain FW measures inside a partial volume free *WM* mask (with and without WMHs) for three different groups (cognitively normal, MCI and AD subjects), selected from the ADNIGO and ADNI2 databases. We show that our FW measures were significantly higher in MCI and AD groups compared to NC when using a WM safe mask. We also show, for the first time, that when WMHs and their penumbrae are removed from the mask, the significant results remained, demonstrating that FW measures are not just a byproduct of WMHs.

## 2. Methods

### 2.1. Study Participants

Two hundred and twenty-six subjects from the ADNIGO and ADNI2 databases passed the necessary quality assurance (QA) phases of the diffusion MRI analysis pipeline (described below). Of those participants, 81 (38 males, 43 females) were cognitively normal (normal control, NC), 103 (69 males, 34 females) had a diagnosis of mild cognitive impairment (MCI) and 42 (25 males, 17 females) had a diagnosis of AD. The Pearson's Chi-squared test revealed a significant difference (*p* = 0.023) in gender between groups. Age range per group was between 67 and 95 years for NCs, between 60 and 95 years for MCIs and between 61 and 97 for ADs. Mean age was 78.46 for NCs, 79.0 for MCIs, and 79.38 for ADs. All participants had good general health, good hearing and seeing abilities, no depression or bipolar condition, no history of alcohol or drug abuse and completed at least six grades of education. Also, NCs had no memory impairment and their CDR was 0. MCI subjects included early and late MCI with impaired memory and a CDR of 0.5, while AD subjects met criteria for dementia and had a CDR between 0.5 and 1 (Petersen et al., 2010). Participants did not suffer from any neurological disorders other than MCI and AD such as brain tumor, multiple sclerosis, Parkinson's disease, or traumatic brain injury. The detailed groups demographics can be seen in Table 1.

**Table 1:** Group demographics of the 226 participants.

	**NC (81)**	**MCI (103)**	**AD (42)**
Age	78.46 (6.11)	79.0 (7.62)	79.38 (7.78)
Gender (M/F)	38/43	69/34	25/17
Education years	16.2 8 (2.74)	15.65 (2.68)	15.07 (2.80)
Ethnicity (H/N/U)	11/70/0	5/98/0	4/37/1
Race (A/B/W/M)	2/4/74/1	2/7/94/0	1/0/40/1
Handedness (R/L)	71/10	92/11	41/1

*M, Male; F, Female; H, Hispanic; N, Not hispanic; U, Unknown; A, Asian; B, Black or African American; W, White; M, More than one race; R, Right; L, Left*.

### 2.2. MRI Data Acquisition

Data used in the preparation of this article were obtained from the Alzheimer's Disease Neuroimaging Initiative (ADNI) database (adni.loni.usc.edu). The ADNI was launched in 2003 as a public-private partnership, led by Principal Investigator Michael W. Weiner, MD. The primary goal of ADNI has been to test whether serial magnetic resonance imaging (MRI), positron emission tomography (PET), other biological markers, and clinical and neuropsychological assessments can be combined to measure the progression of mild cognitive impairment (MCI) and early Alzheimer's disease (AD). For up-to-date information, see www.adni-info.org.

Data for each participant came from the ADNIGO and ADNI2 databases^1^. Of the available MRI images, we used the T1w, diffusion weighted imaging (DWI) and fluid attenuation inversion recovery (FLAIR) scans. The DWI scans were acquired along 41 evenly distributed directions using a b-value of 1,000 s/mm^2^ with a 1.3×1.3×2.7 mm^3^ spatial resolution. The T1w and FLAIR scans were acquired at 1.2×1.05×1.05 and 0.85×0.85×5 mm^3^ spatial resolution, respectively. Data was acquired at 58 different North-American locations.

### 2.3. MRI Processing Pipeline

**Figure 1:**
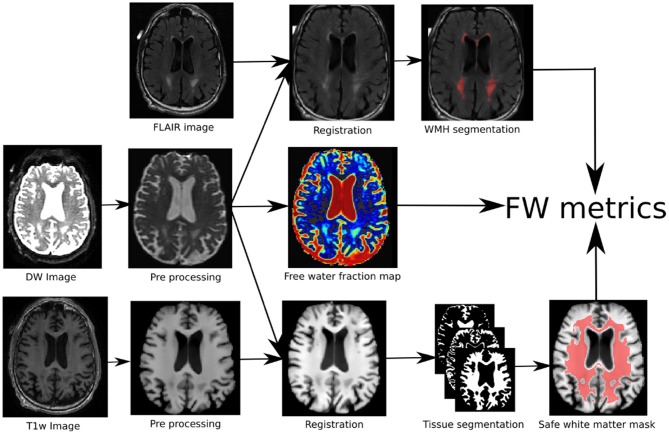
Pipeline of the proposed method: (1) the DWI and T1w images are first preprocessed, (2) the three modalities are co-registered of which (3) are extracted the FW map, the tissue map and the WMHs areas. (4) the combination of the three maps leads to the proposed FW metrics.

The processing pipeline is illustrated in Figure 1. At first, the T1w and DW images were denoised with a non-local means method robust to Rician noise (Descoteaux et al., 2008), followed by an MRI bias field correction performed with ANTs N4 correction tool (Avants et al., 2009). The brain mask (BM) was then processed and the skull was removed using the BEaST brain extraction software (Eskildsen et al., 2012). We referred to these methods as the *preprocessing* step in Figure 1. Then, the T1w and FLAIR images were non-linearly registered to the 1x1x1 up-sampled (using linear interpolation) diffusion space with ANTs registration (Avants et al., 2009). Tissue segmentation was then performed on the transformed T1w scan to obtain a binary map of the CSF, GM, and WM. This was done using ANTs Atropos (Avants et al., 2009). In order to prevent any CSF contamination in regions susceptible to partial volume effect, a “safe WM mask” (*WM*_safe_) was built by combining the following morphological operations on the CSF, WM, GM, and brain binary masks:

(1) $$\begin{array}{r} WM_{\text{safe}}=\left(\left(WM-\left(GM⊕R^{1}\right)\right)-\left(CSF⊕R^{1}\right)\right)\cap \left(BM⊖R^{15}\right), \end{array}$$

where *R*^*n*^ is a 3D structuring element of radius *n*, ⊕ is the dilatation operator, ⊖ is the erosion operator, and ∩ the intersection operator as illustrated in Figure 1. Using the FLAIR and T1w images, a binary map of WMHs was also computed using *volBrain* (Manjón and Coupé, 2016). The WMH maps went through visual QA and none of them were rejected or corrected. Binary dilatation of 2 voxels was applied to the WMHs to avoid partial volume effect contamination and at the same time include the WMH penumbrae. The bi-tensor model proposed by Pasternak et al. (2009) was fit onto the DW signal. The result of this fit is a fraction representing the contribution of unconstrained water to the original signal and a new signal representing the tissue contribution. The fraction of unconstrained water contribution in a voxel is what we commonly call FW volume and the 3D image of this FW volume is called the FW map. The tissue signal is the FW-corrected DWI signal, as it represents the signal without its unconstrained component. The safe white matter mask, the WMH mask, and the FW map were then used to extract the mean FW value (μ*FW*) and the relative FW volume (*rFW*). The *rFW* is the total volume of FW voxels within the safe white matter mask with FW values greater than 0.1, divided by the total volume of the safe white matter mask. The *rFW* was created to minimize the impact of ventricle expansion and whole brain atrophy on the final FW in WM measurement. The 0.1 threshold was defined empirically by observing multiple subjects normal appearing white matter compared to the obvious abnormal values. This enables the *rFW* measurement to discard as much as possible of the background (or noise) FW values.

*rFW*_*m*_ and μ*FW*_*m*_ are defined as such:

(2) $$\begin{array}{r} rFW_{m}=\frac{volume\left(FW_{m}>0.1\right)}{volume\left(m\right)}, \end{array}$$

(3) $$\begin{array}{r} \mu FW_{m}=\bar{FW_{m}}, \end{array}$$

where *m* ∈ {*WM*_safe_, *WMHs, WM*_safe_ − *WMHs*}. All processing was done using a Nextflow (Tommaso et al., 2017) pipeline with all software dependencies bundled in a Singularity container (Kurtzer et al., 2017) ensuring quick and easy reproducibility of the results.

### 2.4. Statistics

A cross-sectional analysis was performed at the first available time point comparing rFW and μFW in NC (*n* = 81), MCI (*n* = 103), and AD (*n* = 42). An analysis of variance (ANOVA) was performed to test for a main effect of diagnostic group followed by a *post-hoc* pairwise Tukey test to assess differences between sub groups (McDonald, 2006). A log transformation was applied to the rFW and μFW metrics to improve normality of the distribution before analyses.

### 2.5. Quality Assurance

Out of all available subjects in ADNI2 and ADNIGO, 239 had at least one time point with all the images required (T1w, DWI, and FLAIR) to go through the processing pipeline. Visual QA was performed on all images of all time points and those with problems impossible to correct (missing brain parts, acquisition artifacts) were rejected. Gradient information was also QA-ed to make sure every DWI image had 41 evenly distributed direction on one single acquisition shell. This first QA pass eliminated 9 subjects bringing the count of subjects with usable data to 230. Visual inspection was performed on brain extraction of T1w and DWI as well as on the non-linear registration of the FLAIR on the T1w and of the T1w on DWI. Every tissue segmentation mask (WM, GM, CSF) as well as the WMH mask was inspected. This second QA pass eliminated 4 subjects, 3 with artifacts in the DWI images causing improbable values in metrics and one with an obviously incorrect T1 brain mask, leaving 226 subjects with usable data for the group analysis.

## 3. Results

**Figure 2:**
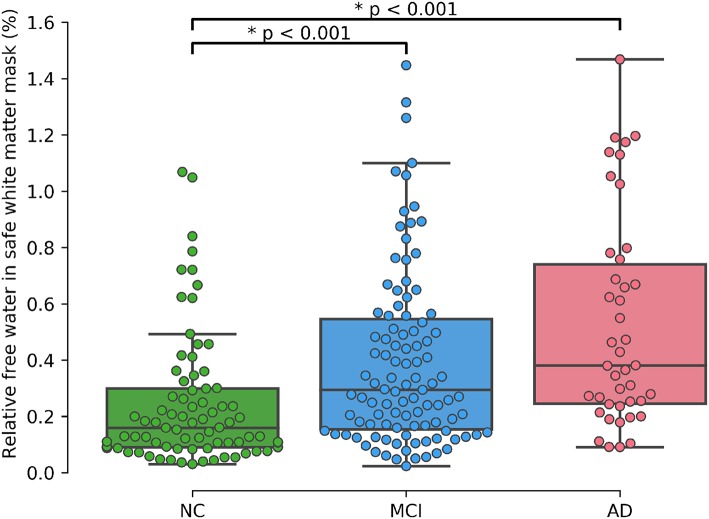
Relative free water (%) in *WM*_safe_ − *WMHs* per group.

As shown in Table 2 results of the initial ANOVA tests show a significant main effect of group membership across all regions of interests. *Post-hoc* Tukey tests show that both *rFW* and μ*FW* are significantly higher in the *WM*_safe mask_ for MCI and AD subjects than for NC subjects whether or not *WMHs* were included as seen in Figure 2. Both *rFW* and μ*FW* in full WM (without partial volume and WMH correction) also differentiate NC from AD and MCI demonstrating that partial volume contaminated measurements can still lead to positive results even though measurements are incorrect. When looking at *rFW* and μ*FW* specifically within the WMH mask we see some significant between-group differences but with lesser effect and neither of them being able to separate both NC-MCI and NC-AD. Finally, the volume of WMH is significantly higher for AD subjects than for NC subjects, highlighting the need for removing WMH from the WM mask since their volume alone differentiates groups.

**Table 2:** The F-statistic obtained from the ANOVA test is displayed in the first column and the rest of the table shows the Tukey *post-hoc* pairwise group differences(on log-scale) with the standard error in parentheses.

	***F*_**(2, 223)**_**	**NC-MCI (SE)**	**NC-AD (SE)**	**MCI-AD (SE)**
*rFW*_*W*_*M*__safe_−*WMHs*_	16.02^***^	–0.52 (0.18)^***^	–0.86 (0.23)^***^	–0.34 (0.20)
μ*FW*_*W*_*M*__safe_−*WMHs*_	13.58^***^	–0.49 (0.17)^***^	–0.70 (0.19)^***^	–0.21 (0.20)
*rFW*_*W*_*M*__safe__	16.48^***^	–0.53 (0.19)^***^	–0.90 (0.22)^***^	–0.36 (0.21)
μ*FW*_*W*_*M*__safe__	14.15^***^	–0.50 (0.17)^***^	–0.74 (0.20)^***^	–0.23 (0.20)
*rFW*_*WM*_	12.79^***^	–0.22 (0.08)^**^	–0.38 (0.11)^***^	–0.16 (0.11)
μ*FW*_*WM*_	12.66^***^	–0.26 (0.09)^***^	–0.40 (0.12)^***^	–0.14 (0.12)
*rFW*_*WMHs*_	4.14^*^	–0.13 (0.07)^*^	–0.14 (0.10)	–0.01 (0.09)
μ*FW*_*WMHs*_	3.80^*^	–0.13 (0.08)	–0.18 (0.10)^*^	–0.05 (0.10)
*WMH*volume	3.11^*^	–0.19 (0.23)	–0.53 (0.27)^*^	–0.33 (0.30)

*The statistical significance (in bold) is shown as*:

* *p < 0.05*,

** *p < 0.01*,

*** *p < 0.001*.

A supplementary ANCOVA test including age and gender as covariates shows that age is highly associated with *rFW* (*p* < 0.001) and gender is marginally associated with *rFW* (*p* = 0.012). After accounting for both gender and age, the significant differences between NC and MCI (*p* < 0.001) as well as between NC and AD (*p* < 0.001) remain.

To assess the viability of measuring FW in all WM (as opposed to bundles) to differentiate groups we visualize the spatial distribution of free water differences between groups. Every T1W image already registered in diffusion space was non-linearly registered to the MNI152 space with the ANTs registration tool (Avants et al., 2009). The resulting transformations were applied to the free water volumes in *WM*_safe_ − *WMHs*. Mean and standard deviation free water volume for each group was computed and used to obtain a z-score volume of each subject compared to each group. These z-score volumes were averaged and thresholded at z ≥ 2 standard deviations to obtain binary group comparison volumes. Only clusters of 10 or more voxels were kept.

**Figure 3:**
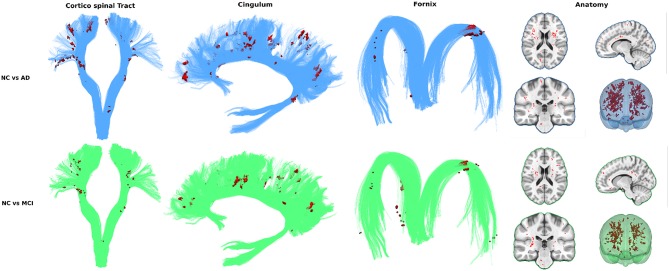
Spatial repartition in free water differences across groups.

In both the NC vs. AD and NC vs. MCI comparisons, voxel clusters showing differences are mostly found in the corticospinal tract (CST) and bundles of the limbic system such as the cingulum and the fornix. Many clusters are also found outside these key AD bundles, generally covering all WM. Figure 3 shows that intensity and location of significant z-score clusters is different when comparing AD or MCI to NC.

## 4. Discussion

A preliminary version of these results was presented at ISMRM 2018 (Dumont et al., 2018) and since then, more studies demonstrating the association of FW in WM and cognitive decline (Maillard et al., 2018; Reas et al., 2018) support the idea that a single whole-brain FW measurement is viable for clinical settings and potential use as a priori outcome measure in clinical trials for diagnostic grouping along the AD continuum.

To achieve that reliable and simple measurement, we identified and overcame three major obstacles (partial volume contamination, WMHs and brain atrophy) to measuring FW content in aging subjects' WM and verified group differentiation with and without each solution. First, as a baseline, FW in whole WM (without correction) was significantly higher in ADs and MCIs than in NCs. Removing partial volume contamination with *WM*_safe_ sharpened group differentiation. Removing WMH and WMH penumbrae slightly decreased differences in groups means while keeping significant differentiation. This can be explained by another result presented demonstrating that WMH volume alone differentiates AD from NC subjects, reinforcing the hypothesis that WMHs and their penumbrae need to be removed to get a relevant measurement of FW in normal appearing WM. We also demonstrated that the group-wise differences of FW content within the WMH lesions was smaller than the group-wise differences of FW content in *WM*_safe_, suggesting that, unlike normal appearing white matter, WMH lesions may have similar underlying pathophysiology across the disease spectrum. Finally, correcting for brain atrophy in aging patients using relative free water volume further sharpened group differentiation. We then visualized the spatial distribution of high FW differences (in *WM*_safe_ − *WMHs*) between groups using high z-scores clusters. These results further strengthened the whole WM measurement idea by showing that while some of the differences are located in bundles known to be associated with AD, the entirety of high z-score clusters globally covers all white matter.

Our new and simple FW measures can be used to increase our understanding of the role of inflammation-associated edema in AD and may aid in the differentiation of healthy subjects from MCI and AD patients. Due to the simplicity of the method and the fast image acquisition time required for the images, these measurements may be particularly useful for clinical settings and can potentially be use as a priori outcome measures in clinical trials.

FW metrics could not differentiate between MCI and AD subjects. This could be the result of the whole white matter measurement not being sensitive enough to differentiate subtle FW differences between MCI and AD. Analyzing FW content along specific WM bundles would be expected to yield more specific results but would also increase complexity by introducing tractography to reconstruct the global WM architecture followed by an automated segmentation of several key WM bundles such as the fornix, cingulum, corpus callosum, and association tracts (arcuate fasciculus, uncinate, inferior longitudinal, and inferior fronto-occipital fasciculus). FW metrics would be analyzed along those bundles, as done in apparent fiber quantification (AFQ) (Yeatman et al., 2012) and tract-profiling (Cousineau et al., 2017). Future work could also include looking at how FW correlates with amyloid beta and tau data available in ADNI to further support the hypothesis that FW is a viable proxy measurement of neuroinflammation.

The FW threshold used to compute *rFW* was defined empirically by observing this particular set of data. Adjustments might be needed to do this analysis on a different database. After the main processing, further tests were done with different thresholds. Group separation remained fairly stable in the 0.1 neighborhood but drops drastically when increasing the threshold past 0.2 due to very low occurrences of these FW values after removing WMHs and partial volume contamination. On the other hand, when lowering the threshold, group separation decreases slowly and stabilizes. This suggests that removing background FW values moderately sharpens group differentiation. An optimal threshold could automatically be found with small increments but it would be specifically tuned for these groups instead of representing the underlying biological phenomenon.

It is important to note that FW metrics used in the current study also have limitations, i.e. they are derived from a bi-tensor model, which is limited to representing a FW compartment and a single fiber population. It is estimated that 66 to 90 percent of brain WM voxels contain at least two fiber populations (Ji et al., 2017; Montal et al., 2018). In those voxels, the estimated contribution of the FW compartment is incorrectly estimated, since some of the signal arising from the fiber populations not fitted to the single fiber tensor may be assigned to the FW compartment. To correct this bias, a FW model accounting for more than one fiber population would need to be used to better fit the signal. While a more sophisticated model would certainly better characterize the information contained in the non-free-water portion of the signal and give more accurate free water indices, these models require multi-shell DWI acquisitions which are unavailable in ADN2 and ADNIGO.

In future works, visualization using z-score clustering could be replaced with a more robust method that takes into account multiple comparisons and cluster-based thresholding such as threshold-free cluster enhancement (Smith and Nichols, 2009) and non-parametric permutation tests (Nichols and Holmes, 2002).

Longitudinal data is available in ADNI2 and ADNIGO but was not analyzed in this study. Future work should make use of this longitudinal data and look into the potential prognostic value of FW values at baseline.

## 5. Conclusion

This study demonstrates that after removing partial volume contamination, removing WMHs and their penumbra and accounting for brain atrophy in elderly, the free water content of healthy looking white matter differentiates MCI and AD groups from healthy subjects. Our method is based on existing DTI-like diffusion data, is atlas free, requires no registration with a reference brain, no PET scan, no tractography, has few tunable parameters, and takes a few minutes only of computation. The method is a simple but powerful approach that may be used clinically or in the context of patient selection and stratification for novel treatments that are aimed at treating or preventing inflammation components of AD using legacy or standard diffusion MRI data. The significant differences of our FW metrics between NC and MCI as well as NC and AD may demonstrate the potential of FW as a tool to study neuroinflammation. We intend to extend this work with analyses of FW metrics in specific white matter bundles and sections of bundles. Also, characterization over time of our new FW metrics in an MCI population could help differentiate those older adults who will remain relatively stable and those who will progress to AD, which has utility for patient selection and stratification of subjects in preclinical stages of AD.

## Data Availability Statement

The image datasets used in this study are publicly available from the ADNI database. Listings of individual subject IDs used in this analysis are available upon request to the corresponding author.

## Ethics Statement

The human data was acquired from the publicly available ADNI database which meet the ethics requirements.

## Author Contributions

MDu: create processing pipeline, process data, design study, write/review text. MR: design study, write/review text, provide biological expertise. P-MJ: write/review text, provide data processing expertise. FM: create processing pipeline, process data, write/review text. J-CH: design study, write/review text, provide diffusion expertise. ZX and MDe: design study, write/review text, provide diffusion imaging expertise. CB: process data, design study, write/review text, provide stats expertise. TS: design study, write/review text, provide neuroinflammation expertise. KV and JG: design study, write/review text, provide MR imaging expertise.

### Conflict of Interest

At the time the work was completed, MDu, MR, P-MJ, FM, J-CH, and MDe were employees at Imeka solutions Inc. and KV, CB, ZX, TS, and JG were employees of Pfizer Inc.

## Footnotes

^1^http://adni.loni.usc.edu/

## References

[B1] Avants B. B. Tustison N. Song G. (2009). Advanced normalization tools (ants). Insight J 2, 1–35.

[B2] Bach M. Laun F. B. Leemans A. Tax C. M. Biessels G. J. Stieltjes B.. (2014). Methodological considerations on tract-based spatial statistics (TBSS). Neuroimage 100, 358–369. 10.1016/j.neuroimage.2014.06.021 24945661

[B3] Brilliant M. Hughes L. Anderson D. Ghobrial M. Elble R. (1995). Rarefied white matter in patients with Alzheimer's. Alzheimer Dis. Assoc. Dis. 9, 39–46. 10.1097/00002093-199505000-00008 7605620

[B4] Brun A. Englund E. (1986a). A white matter disorder in dementia of the Alzheimer type: a pathoanatomical study. Ann. Neurol. 19, 253–262. 10.1002/ana.410190306 3963770

[B5] Brun A. Englund E. (1986b). Brain changes in dementia of Alzheimer's type relevant to new imaging diagnostic methods. Prog. Neuropsychopharmacol. Biol. Psychiatry 10, 297–308. 10.1016/0278-5846(86)90009-6 3492011

[B6] Burns J. M. Church J. A. Johnson D. K. Xiong C. J. Marcus D. Fotenos A. F.. (2005). White matter lesions are prevalent but differentially related with cognition in aging and early Alzheimer disease. Arch. Neurol. 62, 1870–1876. 10.1001/archneur.62.12.1870 16344345

[B7] Chad J. A. Pasternak O. Salat D. H. Chen J. J. (2018). Re-examining age-related differences in white matter microstructure with free-water corrected diffusion tensor imaging. Neurobiol. Aging 71, 161–170. 10.1016/j.neurobiolaging.2018.07.018 30145396 PMC6179151

[B8] Cousineau M. Jodoin P.-M. Garyfallidis E. Côté M.-A. Morency F. C. Rozanski V.. (2017). A test-retest study on Parkinson's PPMI dataset yields statistically significant white matter fascicles. Neuroimage Clin. 16, 222–233. 10.1016/j.nicl.2017.07.020 28794981 PMC5547250

[B9] de la Monte S. M. (1989). Quantitation of cerebral atrophy in preclinical and end–stage Alzheimer's disease. Ann. Neurol. 25, 450–459. 10.1002/ana.410250506 2774485

[B10] de Leeuw F. E. de Groot J. C. Achten E. Oudkerk M. Ramos L. M. Heijboer R.. (2001). Prevalence of cerebral white matter lesions in elderly people: a population based magnetic resonance imaging study: the Rotterdam Scan Study. J. Neurol. Neurosurg. Psychiatry 70, 9–14. 10.1136/jnnp.70.1.9 11118240 PMC1763449

[B11] Debette S. Beiser A. DeCarli C. Au R. Himali J. J. Kelly-Hayes M.. (2010). Association of MRI markers of vascular brain injury with incident stroke, mild cognitive impairment, dementia, and mortality: the framingham offspring study. Stroke 41, 600–606. 10.1161/STROKEAHA.109.570044 20167919 PMC2847685

[B12] DeCarli C. Miller B. L. Swan G. E. Reed T. Wolf P. A. Carmelli D. (2001). Cerebrovascular and brain morphologic correlates of mild cognitive impairment in the national heart, lung, and blood institute twin study. Arch. Neurol. 58, 643–647. 10.1001/archneur.58.4.643 11295996

[B13] Desai M. K. Sudol K. L. Janelsins M. C. Mastrangelo M. A. Frazer E. Bowers W. J. (2009). Triple-transgenic Alzheimer's disease mice exhibit region- specific abnormalities in brain myelination patterns prior to appearance of amyloid and tau pathology. Glia 57, 54–65. 10.1002/glia.20734 18661556 PMC2584762

[B14] Descoteaux M. Wiest-Daesslé N. Prima S. Barillot C. Deriche R. (2008). Impact of rician adapted non-local means filtering on hardi, in International Conference on Medical Image Computing and Computer-Assisted Intervention (New York, NY: Springer), 122–130. 10.1007/978-3-540-85990-1_15 18982597

[B15] Dumont M. Van Dijk K. R. A. Morency F. C. Houde J.-C. Jodoin P.-M. Xie Z. (2018). White matter free water content at different stages of Alzheimer's disease, in Proceedings 26. Annual Meeting International Society for Magnetic Resonance in Medicine, Vol. 26 (Paris), 3753 Available online at: http://archive.ismrm.org/2018/3753.html

[B16] Englund E. (1998). Neuropathology of white matter changes in Alzheimer's disease and vascular dementia. Logo Dementia Geriatr. Cogn. Dis. 9(Suppl. 1), 6–12. 10.1159/000051183 9716238

[B17] Eskildsen S. F. Coupé P. Fonov V. Manjón J. V. Leung K. K. Guizard N.. (2012). Beast: brain extraction based on nonlocal segmentation technique. Neuroimage 59, 2362–2373. 10.1016/j.neuroimage.2011.09.012 21945694

[B18] Galluzzi S. Marizzoni M. Babiloni C. Albani D. Antelmi L. Bagnoli C.. (2016). Clinical and biomarker profiling of prodromal Alzheimer's disease in workpackage 5 of the Innovative Medicines Initiative PharmaCog project: a ‘European ADNI study'. J. Intern. Med. 279, 576–591. 10.1111/joim.12482 26940242

[B19] Hoy A. R. Ly M. Carlsson C. M. Okonkwo O. C. Zetterberg H. Blennow K.. (2017). Microstructural white matter alterations in preclinical Alzheimer's disease detected using free water elimination diffusion tensor imaging. PLoS ONE 12:e0173982. 10.1371/journal.pone.0173982 28291839 PMC5349685

[B20] Ji F. Pasternak O. Liu S. Loke Y. M. Choo B. L. Hilal S.. (2017). Distinct white matter microstructural abnormalities and extracellular water increases relate to cognitive impairment in Alzheimer's disease with and without cerebrovascular disease. Alzheimers Res. Ther. 9, 1–10. 10.1186/s13195-017-0292-4 28818116 PMC5561637

[B21] Jones D. (ed.). (2010). Diffusion MRI: Theory, Methods And Applications. Oxford University Press.

[B22] Kandel B. M. Avants B. B. Gee J. C. McMillan C. T. Erus G. Doshi J.. (2016). White matter hyperintensities are more highly associated with preclinical Alzheimer's disease than imaging and cognitive markers of neurodegeneration. Alzheimers Dement. 4, 18–27. 10.1016/j.dadm.2016.03.001 27489875 PMC4950175

[B23] Kantarci K. Petersen R. Boeve B. Knopman D. (2005). DWI predicts future progression to Alzheimer disease in amnestic mild cognitive impairment. Neurology 64, 902–904. 10.1212/01.WNL.0000153076.46126.E9 15753434 PMC2771335

[B24] Kurtzer G. Sochat V. Bauer M. (2017). Singularity: scientific containers for mobility of compute. PLoS ONE 12:e0177459. 10.1371/journal.pone.0177459 28494014 PMC5426675

[B25] Maier-Hein K. H. Westin C.-F. Shenton M. E. Weiner M. W. Raj A. Thomann P.. (2015). Widespread white matter degeneration preceding the onset of dementia. Alzheimers Dement. 11, 485–493. 10.1016/j.jalz.2014.04.518 25035154 PMC4295016

[B26] Maillard P. Fletcher E. Harvey D. Carmichael O. Reed B. Mungas D. DeCarli C. (2011). White matter hyperintensity penumbra. Stroke 42, 1917–1922. 10.1161/STROKEAHA.110.609768 21636811 PMC3125449

[B27] Maillard P. Fletcher E. Lockhart S. N. Roach A. E. Reed B. Mungas D.. (2014). White matter hyperintensities and their penumbra lie along a continuum of injury in the aging brain. Stroke 45, 1721–1726. 10.1161/STROKEAHA.113.004084 24781079 PMC4102626

[B28] Maillard P. Fletcher E. Singh B. Martinez O. Johnson D. K. Olichney J. (2018). Cerebral white matter free water: a sensitive biomarker of cognition and function. Alzheimers Dement. 14, P1276–P1277. 10.1016/j.jalz.2018.06.1794 PMC6537135 30952798

[B29] Maillard P. Mitchell G. F. Himali J. J. Beiser A. Fletcher E. Tsao C. W.. (2017). Aortic stiffness, increased white matter free water, and altered microstructural integrity: a continuum of injury. Stroke 48, 1567–1573. 10.1161/STROKEAHA.116.016321 28473633 PMC5502744

[B30] Manjón J. V. Coupé P. (2016). volbrain: An online MRI brain volumetry system. Front. Neuroinformatics 10:30. 10.3389/fninf.2016.00030 27512372 PMC4961698

[B31] McDonald J. (2006). Handbook of Biological Statistics. Sparky House Publishing.

[B32] Mito R. Raffelt D. Dhollander T. Vaughan D. N. Tournier J. D. Salvado O.. (2018). Fibre-specific white matter reductions in Alzheimer's disease and mild cognitive impairment. Brain 141, 888–902. 10.1093/brain/awx355 29309541

[B33] Montal V. Vilaplana E. Alcolea D. Pegueroles J. Pasternak O. González-Ortiz S.. (2018). Cortical microstructural changes along the Alzheimer's disease continuum. Alzheimers Dement. 14, 340–351. 10.1016/j.jalz.2017.09.013 29080407

[B34] Nichols T. E. Holmes A. P. (2002). Nonparametric permutation tests for functional neuroimaging: a primer with examples. Hum. Brain Mapp. 15, 1–25. 10.1002/hbm.1058 11747097 PMC6871862

[B35] Nowrangi M. A. Rosenberg P. B. (2015). The fornix in mild cognitive impairment and Alzheimer's disease. Front. Aging Neurosci. 7:1. 10.3389/fnagi.2015.00001 25653617 PMC4301006

[B36] Ofori E. Pasternak O. Planetta P. Li H. Burciu R. Snyder A.. (2015). Free-water within the substantia nigra of Parkinson's disease. Brain 138, 2322–2331. 10.1093/brain/awv136 25981960 PMC4840947

[B37] Pasternak O. Kubicki M. Shenton M. E. (2016). *In vivo* imaging of neuroinflammation in schizophrenia. Schizophr. Res. 173, 200–212. 10.1016/j.schres.2015.05.034 26048294 PMC4668243

[B38] Pasternak O. Shenton M. Westin C.-F. (2012a). Estimation of extracellular volume from regularized multi-shell diffusion MRI. Med. Image Comput. Comput. Assist. Interv. 15, 305–312. 10.1007/978-3-642-33418-4_38 23286062 PMC4039075

[B39] Pasternak O. Sochen N. Gur Y. Intrator N. Assaf Y. (2009). Free water elimination and mapping from diffusion MRI. Magn. Reson. Med. 62, 717–730. 10.1002/mrm.22055 19623619

[B40] Pasternak O. Westin C.-F. Bouix S. Seidman L. J. Goldstein J. M. Woo T.-U. W.. (2012b). Excessive extracellular volume reveals a neurodegenerative pattern in schizophrenia onset. J. Neurosci. 32, 17365–17372. 10.1523/JNEUROSCI.2904-12.2012 23197727 PMC3549332

[B41] Pasternak O. Westin C.-F. Dahlben B. Bouix S. Kubicki M. (2015). The extent of diffusion MRI markers of neuroinflammation and white matter deterioration in chronic schizophrenia. Schizophr. Res. 161, 113–118. 10.1016/j.schres.2014.07.031 25126717 PMC4277709

[B42] Petersen R. C. Aisen P. Beckett L. Donohue M. Gamst A. Harvey D.. (2010). Alzheimer's disease neuroimaging initiative (ADNI): clinical characterization. Neurology 74, 201–209. 10.1212/WNL.0b013e3181cb3e25 20042704 PMC2809036

[B43] Raj D. Yin Z. Breur M. Doorduin J. Holtman I. R. Olah M.. (2017). Increased white matter inflammation in aging-and Alzheimer's disease brain. Front. Mol. Neurosci. 10:206. 10.3389/fnmol.2017.00206 28713239 PMC5492660

[B44] Reas E. T. Hagler D. J. Jr. White N. S. Kuperman J. M. Bartsch H. Wierenga C. E.. (2018). Microstructural brain changes track cognitive decline in mild cognitive impairment. Neuroimage Clin. 20, 883–891. 10.1016/j.nicl.2018.09.027 30290303 PMC6171091

[B45] Sjöbeck M. Haglund M. Englund E. (2005). Decreasing myelin density reflected increasing white matter pathology in azheimer's disease - A neuropathological study. Int. J. Geriatr. Psychiatry 20, 919–926. 10.1002/gps.1384 16163742

[B46] Smith C. D. Chebrolu H. Andersen A. H. Powell D. A. Lovell M. A. Xiong S.. (2010). White matter diffusion alterations in normal women at risk of Alzheimer's disease. Neurobiol. Aging 31, 1122–1131. 10.1016/j.neurobiolaging.2008.08.006 18801597 PMC2873054

[B47] Smith S. M. Jenkinson M. Johansen-Berg H. Rueckert D. Nichols T. E. Mackay C. E.. (2006). Tract-based spatial statistics: voxelwise analysis of multi-subject diffusion data. Neuroimage 31, 1487–1505. 10.1016/j.neuroimage.2006.02.024 16624579

[B48] Smith S. M. Nichols T. E. (2009). Threshold-free cluster enhancement: addressing problems of smoothing, threshold dependence and localisation in cluster inference. Neuroimage 44, 83–98. 10.1016/j.neuroimage.2008.03.061 18501637

[B49] Stebbins G. Murphy C. (2009). Diffusion tensor imaging in Alzheimer's disease and mild cognitive impairment. Behav. Neurol. 21, 39–49. 10.1155/2009/915041 19847044 PMC3010401

[B50] Tommaso P. D. Chatzou M. Floden E. Barja P. Palumbo E. Notredame C. (2017). Nextflow enables reproducible computational workflows. Nat. Biotechnol. 35, 316–319. 10.1038/nbt.3820 28398311

[B51] Yeatman J. D. Dougherty R. F. Myall N. J. Wandell B. A. Feldman H. M. (2012). Tract profiles of white matter properties: automating fiber-tract quantification. PLoS ONE 7:e49790. 10.1371/journal.pone.0049790 23166771 PMC3498174

[B52] Yoshita M. Fletcher E. Harvey D. Ortega M. Martinez O. Mungas D. M.. (2006). Extent and distribution of white matter hyperintensities in normal aging, MCI, and AD. Neurology 67, 2192–2198. 10.1212/01.wnl.0000249119.95747.1f 17190943 PMC3776588

[B53] Zhuang L. Sachdev P. S. Trollor J. N. Reppermund S. Kochan N. A. Brodaty H. (2013). Microstructural white matter changes, not hippocampal atrophy, detect early amnestic mild cognitive impairment. PLoS ONE 8:e58887 10.1371/journal.pone.0058887 23516569 PMC3597581

[B54] Zimmer E. Leuzy A. Benedet A. Breitner J. Gauthier S. Rosa-Neto P. (2014). Tracking neuroinflammation in Alzheimer's disease: the role of positron emission tomography imaging. J. Neuroinflammation 11:120. 10.1186/1742-2094-11-120 25005532 PMC4099095

